# Infusion of Human Albumin on Acute Pancreatitis Therapy: New Tricks for Old Dog?

**DOI:** 10.3389/fphar.2022.842108

**Published:** 2022-06-01

**Authors:** Yifei Ma, Tianao Yan, Fengshuo Xu, Jiachun Ding, Bao Yang, Qingyong Ma, Zheng Wu, Jun Lyu, Zheng Wang

**Affiliations:** ^1^ Department of Hepatobiliary Surgery, The First Affiliated Hospital of Xi’an Jiaotong University, Xi’an, China; ^2^ Department of Surgical Intensive Care Unit, The First Affiliated Hospital of Xi’an Jiaotong University, Xi’an, China; ^3^ Department of Clinical Research, The First Affiliated Hospital of Jinan University, Guangzhou, China; ^4^ School of Public Health, Xi’an Jiaotong University Health Science Center, Xi’an, China; ^5^ Key Laboratory of Environment and Genes Related to Diseases, Xi’an Jiaotong University, Xi’an, China

**Keywords:** albumin infusion, acute pancreatitis, in-hospital mortality, MIMIC-IV, eICU

## Abstract

**Objective:** Human serum albumin (HSA) infusion is a common administration on acute pancreatitis therapy in the Intensive Care Unit (ICU), but its actual association with patients’ outcomes has not been confirmed. The study is aimed to determine whether the in-hospital prognosis of ICU patients with acute pancreatitis could benefit from HSA.

**Methods:** 950 acute pancreatitis patients diagnosed in 2008–2019 were extracted from the MIMIC-IV database as our primary study cohort. The primary outcome was in-hospital mortality. We also performed an external validation with a cohort of 104 acute pancreatitis patients after PSM matching from the eICU database.

**Results:** In MIMIC-IV, 228 acute pancreatitis patients received HSA infusion (Alb group) during their hospitalization, while 722 patients did not (non-Alb group). Patients in the Alb group presented a poorer survival curve than the non-Alb group, while this difference disappeared after PSM or IPTW matching (log-rank test: PSM: *p* = 0.660, IPTW: *p* = 0.760). After including covariates, no association was found between HSA infusion and patients’ in-hospital mortality before and after matching (original cohort: HR: 1.00, 95% CI: 0.66–1.52, *p* = 0.998). HSA infusion also did not benefit patients’ 28-days or ICU mortality, while it was significantly associated with a longer duration of hospital and ICU. In addition, the initial serum albumin levels, infections, the total amount, or the initial timing of infusion did not affect the conclusion. Similarly, in the eICU cohort, HSA infusion was still not a beneficial prognostic factor for patients’ in-hospital prognosis (*p* = 0.087).

**Conclusion:** Intravenous human serum albumin infusion could not benefit acute pancreatitis patients’ in-hospital prognosis and was associated with prolonged hospital and ICU duration.

## Introduction

As the most common gastrointestinal disease requiring emergency hospitalization, acute pancreatitis has an annual incidence of 34 cases per 1,00,000 in high-income countries (Xiao et al., 2016; Boxhoorn et al., 2020). And its incidence is generally considered to be positively correlated with the national sociodemographic index (SDI) (Ouyang et al., 2020). As recent guidelines indicated, gallstones (45%) and alcohol abuse (20%) remain the critical factors in the pathogenesis of acute pancreatitis, which also contributes to the imbalance of incidence between different regions (Roberts et al., 2017; Boxhoorn et al., 2020). Acute pancreatitis is characterized by complex and variable symptoms and various prognoses. Mild cases showed only pancreatic edema, which was often self-limiting and had a good prognosis. In contrast, severe cases (20%) might result in pancreatic necrosis, peritonitis, shock, and systemic multiple organ failure, with 20%–40% mortality (Schepers et al., 2019; Boxhoorn et al., 2020).

The hypoperfusion and intestinal bacterial translocation accompanying systemic inflammatory reactions could lead to irretrievably serious consequences (Hines and Pandol, 2019; Boxhoorn et al., 2020). Therefore, current guidelines clearly state that adequate fluid resuscitation and nutritional support are essential strategies in the initial treatment of acute pancreatitis (Hines and Pandol, 2019). The infusion rate of fluid resuscitation is recommended at 5–10 ml/kg per hour until the patient’s vital signs meet the resuscitation criteria, including heart rate, mean arterial pressure, and urinary output (Working Group IAP/APA Acute Pancreatitis Guidelines, 2013). However, few studies had investigated the type of resuscitation fluid, though several guidelines recommended Ringer’s lactate solution compared with normal saline (Wu et al., 2011; Choosakul et al., 2018; De-Madaria et al., 2018).

Human serum albumin (HSA) has been widely used for volume expansion and correcting hypoalbuminemia in critical care for nearly 70 years worldwide (Vincent et al., 2014). However, clinical evidence for the recommendation of HSA infusion for fluid resuscitation in critical ills remains weak (Das, 2015), and its value in improving hypoalbuminemia is also controversial (Dubois et al., 2006; Finfer et al., 2006). Recently, more and more studies have focused on specific patient groups’ actual survival benefits due to many inappropriate applications of HSA infusion in clinical practice and its reported negative influence on patients’ mortality (Cochrane Injuries Group Albumin Reviewers, 1998; Vincent et al., 2014). Albumin administration is common in clinical practice in acute pancreatitis patients, as in other critical diseases. However, few studies have analyzed whether it has a beneficial impact on acute pancreatitis patients’ outcomes.

Therefore, our study was aimed to determine the effect of human serum albumin infusion on multiple in-hospital outcomes among patients diagnosed with acute pancreatitis through MIMIC-IV (v1.0), a large, retrospective, recently presented, single-center critical care database with a variety of high-quality clinical data from hospital monitoring systems (Johnson et al., 2021). Our study cohort enrolled multifaceted clinical variables of acute pancreatitis patients with ICU (Intensive Care Units) admission to confirm the analysis result. Moreover, some of the potential factors that might affect results, such as patients’ initial serum albumin level during the first 24 h of ICU admission, the total infusion dose or timing of HSA for each patient, and bacterial culture results of patients’ body fluids, were all analyzed in our study. We also analyzed another acute pancreatitis cohort from the eICU database to increase the robustness of our investigation.

## Methods

### Data Source Description

Our data were extracted from MIMIC and eICU databases. The Medical Information Mart for Intensive Care (MIMIC) program is an extensive, single-center, and freely accessible clinical database hosted by the Laboratory for Computational Physiology at the Massachusetts Institute of Technology (MIT) (Johnson et al., 2016; Serpa et al., 2018). The newly released MIMIC-IV (v1.0) in 2021 contained high-quality clinical data for 383,220 patients in Beth Israel Deaconess Medical Center (BIDMC), Boston, from 2008 to 2019 (Johnson et al., 2021). The Philips eICU program is a multi-center database that enrolled 2,00,678 patients from 208 hospitals throughout the continental United States in 2014 and 2015 (Pollard et al., 2018).

### Study Population

**FIGURE 1:**
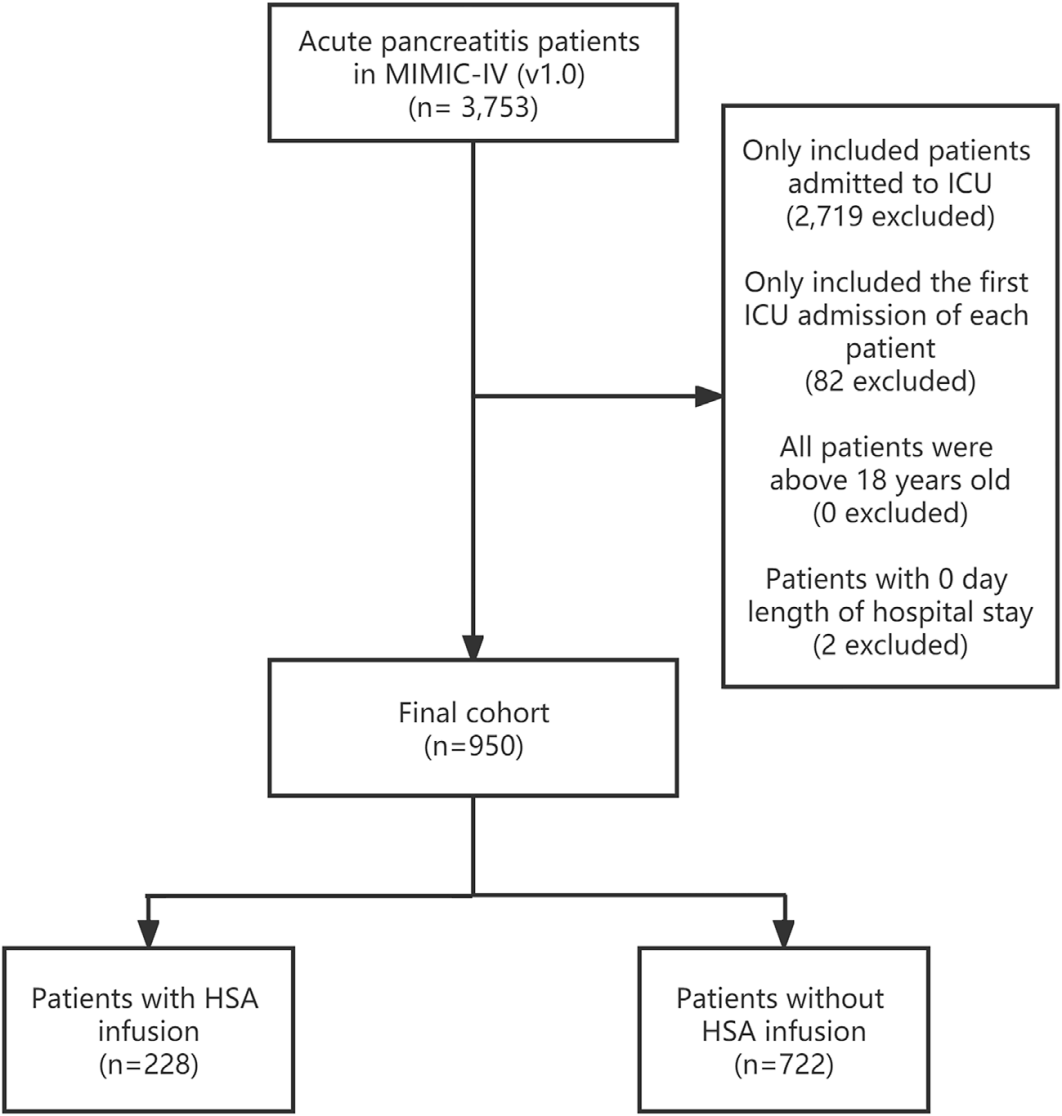
Study sample screening process of 950 acute pancreatitis patients from the MIMIC-IV database. ICU, intensive care unit; HSA, human serum albumin.

Patients whose diagnosis included “acute pancreatitis” were enrolled from both databases in the study. There were 3,753 acute pancreatitis patients in MIMIC-IV totally, and we screened 950 of them only with their first admission to ICU. The sample screening process is shown in Figure 1. Patients younger than 18-years-old or with 0 hospital days were excluded. MIMIC-IV had patients diagnosed with ICD-10 codes (International Classification of Diseases code, version 10), which was different from MIMIC-III (Yang et al., 2020; Johnson et al., 2021; Wu et al., 2021; Zhou et al., 2021), and the number of patients in each diagnosed title is provided in Supplementary Table S1. All 563 patients with acute pancreatitis from eICU met the extraction criteria of MIMIC-IV.

### Variable Extraction and Outcomes

Patients were grouped based on whether they received intravenous HSA infusion during the hospitalization. The total dose (g) of infused HSA and the first infusion time for each patient in MIMIC-IV were also recorded. Other covariates within the first 24 h after ICU admission included the following: age, gender, weight, admission period, Sequential Organ Failure Assessment (SOFA) score, Simplified Acute Physiology Score II (SAPS II) score, application of renal replacement therapy (RRT), mechanical ventilation (MV). The patient’s vital signs were also extracted, including heart rate, mean arterial pressure (MAP), respiratory rate, and temperature (°C). We also enrolled pH, partial pressure of oxygen (pO_2_), partial pressure of carbon dioxide (pCO_2_), lactate level, hemoglobin, platelet counts, white blood cell (WBC) count, serum albumin level, blood urea nitrogen (BUN), and creatinine as laboratory tests in the first 24 h. The comorbidities we extracted as covariates included congestive heart failure (CHF), chronic obstructive pulmonary disease (COPD), liver disease, renal disease, and malignancy. The primary outcome of our study was in-hospital mortality. Secondary outcomes included 28-days mortality, ICU mortality, hospital length of stay (days), and ICU length of stay (days).

### Statistical Analysis

Continuous variables were described by medians with interquartile ranges (IQRs) and compared by *t*-test or Wilcoxon rank-sum test between groups. And we used total number and percentage to present categorical variables and compared the proportions using χ^2^ or Fisher exact tests. As for survival analysis, we used patients’ hospital length of stay (days) as follow-up time and hospital mortality as the primary endpoint. Kaplan-Meier (K-M) survival curve analysis was used to generate curves and the log-rank test to determine statistical differences among groups. Multivariate Cox regression models were performed to determine whether HSA infusion affects patients’ outcomes after the inclusion of varying clinical factors. Moreover, we also applied multivariate linear regression models to analyze hospital outcomes of continuous variables. The selection of covariates included in multivariate analysis considered the data loss rate and its clinical impact on prognosis at the same time. Finally, patients’ admission period, pH, pO_2_, and pCO_2_ were excluded for their irrelevance to patient outcomes or high rate of missing data, while all other covariates were enrolled in multivariate analyses. The amount and percentage of missing data for each covariate in MIMIC-IV are shown in Supplementary Table S2. At the same time, multiple imputations were applied to mitigate the estimation bias caused by missing data and assuming that data were missing randomly in both MIMIC-IV and eICU. The absence rates of covariates included in multivariate analysis in eICU were all less than 20%.

Imbalanced covariates between treatment and control groups might make the results of multivariate analysis less accurate. Propensity score matching (PSM) and propensity score-based inverse probability of treatment weighting (IPTW) methods were applied to minimize the covariate differences between groups (Zhang, 2017; Graffeo et al., 2019). We matched patients in the treatment group to the control group as 1:1 nearest neighbor by estimating the patients’ propensity scores for HSA infusion measurement in PSM (Chen et al., 2019). Moreover, we created two virtual cohorts by weighting each patient through IPTW, which showed a similar distribution of covariates and different administration exposure (Zhang et al., 2018). We also calculated the standardized mean differences (SMD) and performed χ^2^ or *t*-tests before and after matching to examine the effects of PSM and IPTW. Due to insufficient covariates, small sample size, and significant differences in the number of patients between groups of the eICU cohort, We used PSM matched eICU cohort as an external validation of patients from MIMIC-IV. The baseline covariates and SMDs of patients from eICU are presented in Supplementary Table S3 after being PSM matched.

All our patients’ data from the database were extracted in SQL (Structured Query Language), and all statistical analyses were performed by Rstudio software (v3.6.3). Two-sided *p* < 0.05 was considered statistically significant.

## Results

### Baseline Characteristics

The entire study cohort included 950 patients diagnosed with acute pancreatitis between 2008 and 2019, of whom 228 received human serum albumin infusion during hospitalization (Alb group), and the remaining did not (non-Alb group). Patients’ baseline characteristics of the entire and different treatment groups are shown in Table 1. In general, the Alb group was more likely to receive RRT (12.7% vs. 4.6%; *p* < 0.001) and mechanical ventilation (56.6% vs. 27.6%; *p* < 0.001) than the non-Alb group. Additionally, Alb group had significantly higher SOFA [9 (5–13) vs. 4 (2–7); *p* < 0.001] and SAPS II scores [43 (34–57) vs. 30 (22–42); *p* < 0.001]. More patients in the Alb group were found with liver disease (40.8% vs. 24.2%; *p* < 0.001) and malignancy (12.7% vs. 6.4%; *p* = 0.003) than the non-Alb group. As for vital signs, patients in the Alb group had faster heart rate [100 (86–112 bpm) vs. 92 (78–106 bpm); *p* < 0.001], faster respiratory rate [21 (18–25 bpm) vs. 20 (17–23 bpm); *p* = 0.001] and lower mean arterial pressure [76 (71–86 mmHg) vs. 82 (74–92 mmHg); *p* < 0.001]. In laboratory tests, we also found significant differences between the two groups. There were lower pH, lower serum albumin, lower hemoglobin levels, higher pO_2_, higher lactate, higher WBC, higher Bun, and higher creatinine levels in the Alb group.

**TABLE 1:** Baseline characteristics of the included patients from the MIMIC-IV database.

Covariates	MIMIC-IV (*n* = 950)				
	All patients	Non-alb	Alb	*p* value	SMD
N	950	722	228		
Age	58 (46–71)	57 (45–71)	58 (48–72)	0.328	0.074
Male (%)	544/950 (57.3)	413/722 (57.2)	131/228 (57.5)	1.000	0.005
Weight (kg)	81.0 (70.0–97.8)	80.7 (68.9–97.0)	81.4 (71.2–99.5)	0.150	0.073
Admission period, *n* (%)				0.107	0.127
2008–2013	607/950 (63.9)	472/722 (65.4)	135/228 (59.2)		
2014–2019	343/950 (36.1)	250/722 (34.6)	93/228 (40.8)		
Interventions, *n* (%)					
RRT use (1st 24 h)	62/950 (6.5)	33/722 (4.6)	29/228 (12.7)	<0.001	0.293
MV use (1st 24 h)	328/950 (34.5)	199/722 (27.6)	129/228 (56.6)	<0.001	0.615
Severity					
SOFA score	5 (3–9)	4 (2–7)	9 (5–13)	<0.001	0.834
SAPS II score	33 (23–45)	30 (22–42)	43 (34–57)	<0.001	0.803
Comorbidities, *n* (%)					
CHF	183/950 (19.3)	143/722 (19.8)	40/228 (17.5)	0.510	0.058
COPD	205/950 (21.6)	152/722 (21.1)	53/228 (23.2)	0.542	0.053
Liver disease	268/950 (28.2)	175/722 (24.2)	93/228 (40.8)	<0.001	0.359
Renal disease	167/950 (17.6)	121/722 (16.8)	46/228 (20.2)	0.279	0.088
Malignancy	75/950 (7.9)	46/722 (6.4)	29/228 (12.7)	0.003	0.217
Vital signs					
Heart rate (bpm)	93 (80–107)	92 (78–106)	100 (86–112)	<0.001	0.399
MAP (mmHg)	81 (73–91)	82 (74–92)	76 (71–86)	<0.001	0.352
Respiratory rate (bpm)	20 (17–24)	20 (17–23)	21 (18–25)	0.001	0.284
Temperature (°C)	36.9 (36.6–37.3)	36.9 (36.7–37.3)	36.9 (36.6–37.2)	0.070	0.145
Laboratory tests					
pH	7.37 (7.29–7.43)	7.37 (7.30–7.43)	7.36 (7.26–7.41)	0.016	0.155
pO_2_ (mmHg)	81 (51–135)	79 (49–127)	87 (57–167)	0.007	0.222
pCO_2_ (mmHg)	39 (33–45)	39 (33–46)	39 (33–44)	0.711	0.017
Lactate level (mmol/L)	1.7 (1.2–2.7)	1.6 (1.2–2.5)	2.1 (1.4–3.5)	<0.001	0.337
Hemoglobin (×10^12^/L)	11.1 (9.5–12.7)	11.3 (9.7–12.8)	10.7 (9.0–12.6)	0.005	0.177
Platelet (×10^9^/L)	188 (129–268)	191 (136–270)	172 (117–264)	0.021	0.122
WBC (×10^9^/L)	12.2 (8.6–17.3)	12.1 (8.4–16.5)	13.0 (9.0–19.5)	0.003	0.270
Albumin (g/dl)	3.1 (2.6–3.5)	3.1 (2.7–3.6)	2.8 (2.4–3.3)	<0.001	0.449
BUN (mg/dl)	19 (12–35)	17 (11–31)	26 (17–48)	<0.001	0.358
Creatinine (mg/dl)	1.1 (0.7–1.8)	1.0 (0.7–1.7)	1.4 (0.9–2.6)	<0.001	0.238

Alb, human serum albumin infusion; SMD, standardized mean differences; RRT, renal replacement therapy; MV, mechanical ventilation; SOFA, sequential organ failure assessment; SAPS II, simplified acute physiology score II; CHF, congestive heart failure; COPD, chronic obstructive pulmonary disease; MAP, mean arterial pressure; pO_2_, partial pressure of oxygen; pCO_2_, partial pressure of carbon dioxide; WBC, white blood cell; BUN, blood urea nitrogen.

### Primary Outcome

**FIGURE 2:**
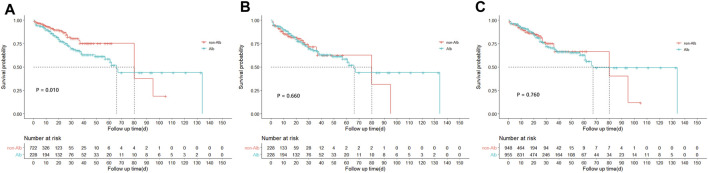
Kaplan-Meire survival curve analysis between treatment groups before and after matching from the MIMIC-IV database. **(A)** Survival curves of hospital mortality in acute pancreatitis patients between treatment groups before matching from the MIMIC-IV database. **(B)** Survival curves of hospital mortality in acute pancreatitis patients between treatment groups after PSM matching from the MIMIC-IV database. **(C)** Survival curves of hospital mortality in acute pancreatitis patients between treatment groups after IPTW matching from the MIMIC-IV database. PSM, propensity score matching; IPTW, inverse probability of treatment weighing.

**FIGURE 3:**
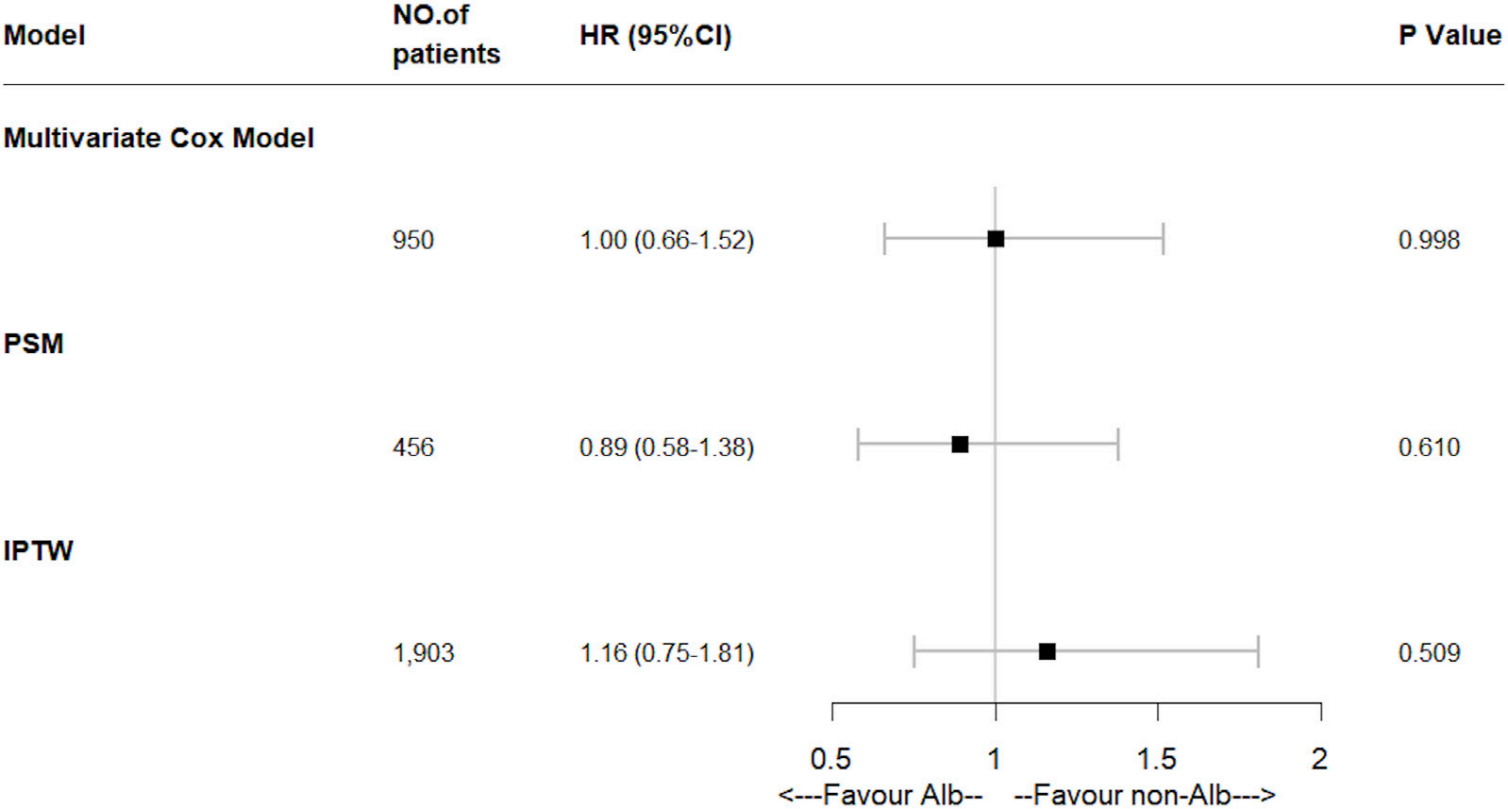
Effect of human serum albumin infusion on primary outcome in acute pancreatitis patients from the MIMIC-IV database before and after matching through multivariate Cox regressions. HR, hazard ratio; PSM, propensity score matching; IPTW, inverse probability of treatment weighing.

A total of 125 (13.2%) in-hospital deaths occurred in the entire study cohort, including 67 (29.4%) in the Alb group and 58 (8.0%) in the non-Alb group. The total median hospital length of stay was 11 (6–20) days, and 22 (13–38) days in the Alb group, 9 (5–15) days in the non-Alb group, respectively. After the Kaplan-Meier survival curve analysis, a significant survival difference (*p* = 0.010) was found between the two groups (Figure 2A). The Alb group tended to have worse in-hospital survival status than the non-Alb group. Then the independence of HSA infusion as a prognostic factor in patients’ hospital mortality was analyzed using multivariate Cox regression models. However, we found no association (HR: 1.00, 95% CI: 0.66–1.52, *p* = 0.998) between HSA infusion and hospital mortality of acute pancreatitis patients admitted to ICU (Figure 3).

Furthermore, to mitigate the estimation bias caused by imbalanced covariates between different treatment groups, we performed PSM and IPTW methods. The imbalance of covariates between groups was significantly reduced after both matches (Supplementary Table S4). In these two matched study cohorts, differences between groups of the K-M survival curves disappeared through log-rank tests (Figure 2B, PSM: *p* = 0.660; Figure 2C, IPTW: *p* = 0.760). Moreover, the multivariate Cox regression model showed similar results as the original cohort after being matched (Figure 3). To increase the robustness of this study, we also performed multivariate analyses in original and matched cohorts of patients without missing data, and the results remained similar. (Supplementary Tables S5, S6).

### Secondary Outcomes

**FIGURE 4:**
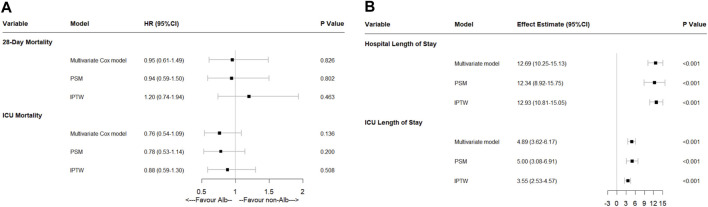
Effect of human serum albumin infusion on secondary outcomes in acute pancreatitis patients from the MIMIC-IV database before and after matching through multivariate analyses. **(A)** Effect of human serum albumin infusion on 28-days mortality and ICU mortality in acute pancreatitis patients from the MIMIC-IV database before and after matching through multivariate Cox regressions. **(B)** Effect of human serum albumin infusion on hospital and ICU length of stays in acute pancreatitis patients from the MIMIC-IV database before and after matching through multivariate linear regressions. HR, hazard ratio; ICU, intensive care unit; PSM, propensity score matching; IPTW, inverse probability of treatment weighing.

Human serum albumin infusion was also found no association with 28-days mortality and ICU mortality by multivariate Cox regressions (28-days mortality: HR: 0.95, 95% CI: 0.61–1.49, *p* = 0.826; ICU mortality: HR: 0.76, 95% CI: 0.54–1.09, *p* = 0.136). Moreover, after multivariate linear regressions, there were significant associations between HSA infusion and more extended hospital and ICU length of stay in ICU admitted acute pancreatitis patients (*p* < 0.001). To verify the results, we also performed PSM and IPTW methods. After being matched, the results were similar (Figure 4).

### Subgroup and Sensitivity Analyses

To investigate the effect of HSA infusion on patients’ outcomes with different initial serum albumin levels, patients in the MIMIC-IV cohort were divided into four subgroups by first measured serum albumin level after ICU admission: <2.5 g/dl group, 2.5–3.0 g/dl group, 3.0–3.5 g/dl group, and ≥3.5 g/dl group. K-M survival analyses of each subgroup are shown in Supplementary Figure S1, while multivariate Cox regressions were also performed, as shown in Supplementary Figure S2. Interestingly, HSA infusion negatively influenced patients with 3.0–3.5 g/dl serum albumin (HR: 8.17, 95% CI: 2.01–33.14, *p* = 0.003, *n* = 250), while no association with hospital mortality in other subgroups had been found (Supplementary Figure S2). After being matched by different methods, we repeated the analysis and got similar results (Supplementary Tables S7–S10). It seemed that HSA infusion could not benefit patients’ outcomes. This result was independent of the patients’ initial serum albumin level.

Bacteraemia and secondary infection are common in acute pancreatitis patients and are essential factors leading to more severe disease (Besselink et al., 2009). To determine whether patients subgroup with definite infection would benefit from HSA infusion, we performed a subgroup analysis of patients with positive blood or peritoneal fluid culture. In the MIMIC-IV cohort, 161 patients with acute pancreatitis had positive blood or peritoneal fluid culture during hospitalization. We performed K-M survival analyses and multivariate Cox regressions, and HSA infusion was still not associated with in-hospital mortality in acute pancreatitis patients with definite infection (Supplementary Figure S3; Supplementary Table S11).

Additionally, we also investigated the effect of different total doses of HSA infusion on hospital outcomes as sensitivity analyses. According to clinical practice, patients with HSA infusion were divided into two subgroups (infusion dose < 100 g; ≥100 g) and compared against the non-Alb group, respectively. According to our results, albumin administration did not influence the primary outcome of patients with acute pancreatitis in either infusion volume subgroup (Supplementary Figure S4; Supplementary Tables S12, S13).

There are two overlapping peaks of death in patients with acute pancreatitis. The first peak was clinically characterized by SIRS or organ failure, and the second was intraperitoneal infection or necrosis of the pancreas or peripancreatic tissue (Gardner, 2021). To investigate the effect of HSA infusion on the prognosis of patients with acute pancreatitis at different clinical stages, we divided patients receiving HSA infusion into within 72 h and beyond according to the time interval between admission to ICU and initial infusion. The effect of early HSA infusion (within 72 h of admission to the ICU) on prognosis compared with those who did not receive HSA infusion was studied, as shown in Supplementary Figure S5 and Supplementary Table S14. Through multivariate analysis, early infusion of HSA was also not associated with hospital outcomes in patients with acute pancreatitis.

### External Validation With Propensity Score-Matched eICU Cohort

We also validated our results with acute pancreatitis patients extracted from the eICU database. After being PSM matched, 104 acute pancreatitis patients with ICU admission were enrolled (52 in the Alb group and 52 in the non-Alb group). Baseline characteristics of these patients after PSM are presented in Supplementary Table S3, as mentioned. Though there was a survival difference between treatment groups in the K-M survival curves analysis (*p* = 0.037), HSA infusion still did not influence multiple in-hospital outcomes of acute pancreatitis patients after multivariate analyses (*p* = 0.087) (Supplementary Figures S6, S7).

## Discussion

In general, our research is the first clinical investigation concentrating on the role of human serum albumin infusion in the hospital outcomes of patients diagnosed with acute pancreatitis since 2008. Through a retrospective cohort of 950 contemporary acute pancreatitis patients from the MIMIC-IV database, our research showed that the infusion of HSA was not associated with ICU acute pancreatitis patients’ in-hospital mortality but significantly associated with their more extended hospital and ICU stays. This result was independent of whether the patient had a positive bacterial culture result. In subsequent subgroup analyses, HSA infusion still did not affect the patients’ prognosis with significant hypoalbuminemia (<2.5 g/dl) while even tended to have an adverse effect on the group of patients with near-normal (3.0–3.5 g/dl) initial serum albumin levels. A cohort from the eICU database partially supported these results above after being PSM matched (104 patients).

Acute pancreatitis is characterized by local, systemic inflammatory, and immune responses, leading to organ failure and even death in severe cases. Subsequent fluid extravasation in the third space is one of the critical reasons for the severity of the disease (van Dijk et al., 2017; Hines and Pandol, 2019; Boxhoorn et al., 2020). In current guidelines, adequate fluid resuscitation is considered an essential step of the initial treatment in severe acute pancreatitis patients (Boxhoorn et al., 2020). As indicated, the goal-directed therapy advised a resuscitation rate of 5–10 ml/kg/h to avoid the potentially detrimental influence that improper fluid replacement might cause (Crockett et al., 2018). While as for the type of fluid, study evidence with high confidence is still scarce. Only several RCTs with a small sample size concluded that Ringer’s lactate solution had an unconfirmed benefit in reducing the chance of SIRS (systemic inflammatory response syndrome) and C-relative protein concentrations compared with normal salina (Wu et al., 2011; Working Group IAP/APA Acute Pancreatitis Guidelines, 2013; Iqbal et al., 2018; Choosakul et al., 2018; De-Madaria et al., 2018).

Though the value of colloids was not confirmed in the therapy of acute pancreatitis (van Dijk et al., 2017), it has been demonstrated that HSA required less fluid than crystalloid solutions to provide effective fluid resuscitation and might reduce the mortality in critically ill patients (Vincent et al., 2014; Gomez et al., 2021). On the other hand, some studies also showed that hypoalbuminemia negatively influenced acute pancreatitis patients’ prognosis significantly (Finfer et al., 2006; Hong et al., 2017). For the reasons above, the doctors were accustomed to applying the albumin infusion to increase colloidal osmotic pressure and improve hypoalbuminemia in clinical practice. However, the actual association between human serum albumin infusion and acute pancreatitis patients’ prognosis has not been confirmed by clinical studies so far.

To confirm the influence of HSA infusion on acute pancreatitis, we designed the research. Our primary study cohort was extracted from MIMIC-IV (v1.0), published on 16 March 2021. MIMIC-IV was a newly-updated version of MIMIC-III, which had been improved in numerous aspects (Johnson et al., 2021). In MIMIC-IV, the patients’ data from 2008 to 2019 could better reflect the current diagnosis and treatment of diseases and provide better suggestions for the current clinical practice. Our research also used acute pancreatitis patients’ data from the eICU database. The eICU Collaborative Research Database (v2.0) contained clinical data of patients with ICU admission from 208 hospitals in 2014 and 2015 (Pollard et al., 2018). In our study process, we applied the MIMIC-IV cohort as our primary analysis group, while the eICU cohort after PSM matching was applied as an external verification.

In our study, patients in the HSA infusion group showed a more severe disease state than the other group, which could be indicated by discrepant parameters such as higher SOFA, SAPS II scores, lower serum albumin level, and lower mean arterial pressure. This phenomenon was consistent with the clinical decision strategy often made by doctors previously analyzed. The Alb group showed a poorer prognosis in K-M survival analyses, and this survival difference disappeared after balancing covariates between treatment groups by PSM or IPTW methods. Furthermore, after including covariates from multiple clinical aspects of each patient, multivariate Cox regressions still showed no correlation between HSA infusion and patients’ in-hospital prognosis before and after matching. In addition, through multivariate linear regressions, we found that intravenous HSA infusion was associated with a longer hospital length of stay and ICU duration. It seemed that HSA infusion could not benefit acute pancreatitis patients’ prognosis. This is consistent with previous studies (Myburgh et al., 2007; Roberts et al., 2011; Caironi et al., 2014; Patel et al., 2014; Uhlig et al., 2014; Thevenot et al., 2015; Eljaiek et al., 2017; Leao et al., 2019). Since the meta-analysis study of increased mortality rates in patients who received albumin solutions was first reported in 1998 (Cochrane Injuries Group Albumin Reviewers, 1998), more and more well-controlled RCTs have concentrated on the actual benefit of human serum albumin in specific patient groups. One of the most influential prospective studies, published in 2014, was a multi-center trial of 1,818 patients with severe sepsis, which concluded that the addition of albumin did not improve the 28- or 90-days mortality compared with crystalloids alone (Caironi et al., 2014). Another trial, including 193 cirrhotic patients with infection other than SBP (spontaneous bacterial peritonitis) in 2015, also negated the benefit of albumin infusion in overall patient survival and improvement of renal failure (Thevenot et al., 2015). The plausibility of our results was strongly supported by many prospective studies that had concluded similar opinions with other specific patient groups in recent years (Myburgh et al., 2007; Roberts et al., 2011; Patel et al., 2014; Uhlig et al., 2014; Eljaiek et al., 2017; Leao et al., 2019). We also performed an external validation with a cohort of 104 well-matched acute pancreatitis patients from the eICU database to strengthen our conclusion. Similar to the results of the MIMIC-IV cohort, HSA infusion continued to have no beneficial effect on primary and secondary outcomes in patients with acute pancreatitis through K-M survival analysis and multivariate Cox regressions.

To further support our results, we also conducted well-developed subgroup and sensitivity analyses. The patient’s initial serum albumin level was likely to influence the study results from clinical practice, and we performed subgroup analyses for patients with different first measured serum albumin levels after ICU admission. According to our results, even among acute pancreatitis patients with obvious hypoalbuminemia (<2.5 g/dl), HSA infusion still had no statistically significant advantage on patients’ outcomes before and after matching. This finding was consistent with a large meta-analysis study published in recent years that there was no evidence that albumin infusion improved prognosis in critically ill patients with baseline hypoalbuminemia (Patel et al., 2014). Moreover, compared with other subgroups, HSA infusion had a negative effect on the prognosis of patients whose initial serum albumin level was 3.0–3.5 g/dl. This phenomenon might be explained by the body’s self-compensation mechanism, suggesting that albumin infusion might be even more discouraged in acute pancreatitis patients with albumin levels near normal. It has been proved that early bacteremia and secondary pancreatic or peripancreatic necrosis might result in sepsis with a poor prognosis in acute pancreatitis patients (Besselink et al., 2009). Thus, we performed a subgroup analysis of patients with positive blood or peritoneal fluid bacterial cultures, and the results were robust to the primary analysis. As mentioned above, the role of albumin infusion in sepsis patients has been extensively studied in recent years. The current consensus was that the benefit of albumin in improving the prognosis of patients with sepsis relative to crystalloids remained unclear (Gotts and Matthay, 2016), which was consistent with our results. Our study also considered the possible impact of the total HSA infusion dosage on the results as a sensitivity analysis. According to the results, HSA infusion was not a beneficial factor, regardless of the total dose.

The initial management of acute pancreatitis included not only adequate fluid resuscitation but also effective nutritional support (Boxhoorn et al., 2020). Current guidelines mainly recommend enteral feeding because of its beneficial role in nourishing the intestinal barrier, preventing bacterial translocation, and reducing the probability of SIRS when compared with conventional parenteral nutrition (Petrov et al., 2008; Capurso et al., 2012; Hines and Pandol, 2019; Boxhoorn et al., 2020). On the other hand, serum albumin level was also an important indicator in evaluating the nutritional status of patients (Hines and Pandol, 2019). In combination with our findings, it was not difficult to conclude that due to the irrelevance of intravenous HSA infusion to patient prognosis, our study might emphasize the importance and necessity of enteral nutritional support for patients with acute pancreatitis from another perspective.

There were still several limitations in our research. First, the estimation bias was unavoidable as a retrospective study due to complex confounding factors in actual clinical treatment that could not be considered, though we had already significantly reduced the bias by several adjustments and a well-developed subgroup analysis. Large-scale, well-controlled RCTs are still desperately required to reach a more convincing conclusion. Secondly, due to the limitations of the MIMIC-IV and eICU database, the clinical indicators reflecting the possible benefits of drug administration were still insufficient, resulting in the possibility of neglecting the potential beneficial effects of HSA infusion for patients with acute pancreatitis. For example, previous studies had reported significant hemodynamic advantages of HSA infusion in patients with sepsis, although improvement in patient outcomes was also not observed (Caironi et al., 2014). In addition, the MIMIC-IV database was still unable to obtain data on patients’ out-of-hospital survival status due to inadequate follow-up time (Johnson et al., 2021), which might result in our study ignoring the possible positive influence of HSA infusion on patients’ long-term survival.

## Conclusion

In conclusion, intravenous human serum albumin infusion could not benefit acute pancreatitis patients’ in-hospital prognosis and was associated with prolonged hospital and ICU duration. This conclusion remained robust in patient subgroups with significant hypoalbuminemia (<2.5 g/dl), positive bacterial cultures in blood or peritoneal fluid, different initial infusion times, and total infusion doses.

## Data Availability

The datasets presented in this study can be found in online repositories. The names of the repository/repositories and accession number(s) can be found below: The datasets generated and analyzed during the current study are available in the MIMIC-IV and eICU databases, https://physionet.org/content/mimiciv/1.0/ and https://physionet.org/content/eicu-crd/2.0/.
