# Supplementary material for: Infusion of Human Albumin on Acute Pancreatitis Therapy: New Tricks for Old Dog?
Source: Front Pharmacol. 2022 Jun 1;13:842108. doi: 10.3389/fphar.2022.842108 (PMC9198420; doi:10.3389/fphar.2022.842108)
Supplement: Supplementary file 1 [file DataSheet1.docx]

Infusion of human albumin on acute pancreatitis therapy: new tricks for old dog?

Yifei Ma, MD^1,2^, Tianao Yan, MD^1,2^, Fengshuo Xu, MD^3,4^, Jiachun Ding, MD^1,2^, Bao Yang, MD^1,2^, Qingyong Ma, Ph.D^1^, Zheng Wu, Ph.D^1^, Jun Lyu, Ph.D^3^, Zheng Wang, Ph.D^1,2,5^

^1^Department of Hepatobiliary Surgery, The First Affiliated Hospital of Xi'an Jiaotong University, 277 West Yanta Road, Xi'an, 710061, People's Republic of China

^2^Department of Surgical Intensive Care Unit, The First Affiliated Hospital of Xi'an Jiaotong University, 277 West Yanta Road, Xi'an, 710061, People's Republic of China

^3^Department of Clinical Research, The First Affiliated Hospital of Jinan University, 613 West Huangpu Avenue, Guangzhou, 510630, People's Republic of China

^4^School of Public Health, Xi'an Jiaotong University Health Science Center, 76 West Yanta Road, Xi'an, 710061, People's Republic of China

^5^Key Laboratory of Environment and Genes Related to Diseases, Xi'an Jiaotong University, 28 West Xianning Road, Xi'an, 710049, People's Republic of China

**Author details**

First author: Yifei Ma, Resident Doctor in Hepatobiliary Surgery, The First Affiliated Hospital of Xi'an Jiaotong University. Tel: (+86) 029-85324695, Email: mayiphy4628@stu.xjtu.edu.cn, ORCID: 0000-0001-5301-5562, Address: 277 Yanta Western Rd., Xi'an, 710061, Shaanxi Province, People's Republic of China,

*Co-corresponding author: Dr. Jun Lyu, Chief of Clinical Research Department, The First Affiliated Hospital of Jinan University, Tel: (+86) 13379060167, Email: [lyujun2020@jnu.edu.cn,](mailto:lyujun2020@jnu.edu.cn,) ORCID: 0000-0002-2237-8771, Address: 613 West Huangpu Avenue, Guangzhou, 510630, Guangdong Province, People's Republic of China,

**Corresponding author: Zheng Wang, Prof, Ph.D., Professor in Hepatobiliary Surgery and Chief of Surgical Intensive Care Unit department, The First Affiliated Hospital of Xi'an Jiaotong University, Tel: (+86) 029-85324695, Email: zheng.wang11@mail.xjtu.edu.cn, ORCID: 0000-0002-0490-466X, Address: 277 Yanta Western Rd., Xi'an, 710061, Shaanxi Province, People's Republic of China.

**Table S1: Number of patients in each diagnosed title of 950 acute pancreatitis patients from the MIMIC-IV database in ICD standard**

| Diagnosis | ICD code | ICD version | No.of  patients |
| --- | --- | --- | --- |
| Acute pancreatitis | 5770 | 9 | 685 |
| Acute pancreatitis without necrosis or infection, unspecified | K8590 | 10 | 103 |
| Biliary acute pancreatitis without necrosis or infection | K8510 | 10 | 46 |
| Alcohol induced acute pancreatitis without necrosis or infection | K8520 | 10 | 33 |
| Alcohol induced acute pancreatitis with uninfected necrosis | K8521 | 10 | 22 |
| Acute pancreatitis with uninfected necrosis, unspecified | K8591 | 10 | 16 |
| Other acute pancreatitis without necrosis or infection | K8580 | 10 | 14 |
| Biliary acute pancreatitis with infected necrosis | K8511 | 10 | 9 |
| Biliary acute pancreatitis with uninfected necrosis | K8512 | 10 | 9 |
| Idiopathic acute pancreatitis without necrosis or infection | K8500 | 10 | 4 |
| Drug induced acute pancreatitis without necrosis or infection | K8530 | 10 | 3 |
| Alcohol induced acute pancreatitis with infected necrosis | K8522 | 10 | 2 |
| Other acute pancreatitis with uninfected necrosis | K8581 | 10 | 2 |
| Drug induced acute pancreatitis with uninfected necrosis | K8502 | 10 | 1 |
| Idiopathic acute pancreatitis with infected necrosis | K8531 | 10 | 1 |

ICD: International Classification of Diseases

**Table S2: The information of missing data in the variables of interest of 950 acute pancreatitis patients from the MIMIC-IV database**

| Variables | Missing, n (%) |
| --- | --- |
| Age | 0 (0) |
| Gender | 0 (0) |
| Weight (kg) | 30 (3.1) |
| Admission period | 0 (0) |
| Interventions |  |
| RRT use (1^st^ 24h) | 0 (0) |
| MV use (1^st^ 24h) | 0 (0) |
| Severity |  |
| SOFA score | 0 (0) |
| SAPS II score | 0 (0) |
| Comorbidities |  |
| CHF | 0 (0) |
| COPD | 0 (0) |
| Liver disease | 0 (0) |
| Renal disease | 0 (0) |
| Malignancy | 0 (0) |
| Vital signs |  |
| Heart rate (bpm) | 2 (0.2) |
| MAP (mmHg) | 2 (0.2) |
| Respiratory rate (bpm) | 2 (0.2) |
| Temperature (°C) | 12 (1.2) |
| Laboratory tests |  |
| pH | 224 (23.5) |
| pO2 (mmHg) | 267 (28.1) |
| pCO2 (mmHg) | 268 (28.2) |
| Lactate level (mmol/L) | 193 (20.3) |
| Hemoglobin (×10^12^/L) | 5 (0.5) |
| Platelet (×10^9^/L) | 4 (0.4) |
| WBC (×10^9^/L) | 4 (0.4) |
| Albumin (g/dL) | 140 (14.7) |
| BUN (mg/dL) | 5 (0.5) |
| Creatinine (mg/dL) | 3 (0.3) |

RRT renal replacement therapy, MV mechanical ventilation, SOFA Sequential Organ Failure Assessment, SAPS II Simplified Acute Physiology Score II, CHF congestive heart failure, COPD chronic obstructive pulmonary disease, MAP mean arterial pressure, pO_2_ partial pressure of oxygen, pCO_2_ partial pressure of carbon dioxide, WBC white blood cell, BUN blood urea nitrogen

| **Table S3: Baseline characteristics of acute pancreatitis patients from the eICU database after PSM matching** | | | | | |
| --- | --- | --- | --- | --- | --- |
| Covariates | eICU (n = 104) | | | | |
|  | All | non-Alb | Alb | P value | SMD |
| n | 104 | 52 | 52 |  |  |
| Age | 55 (44-67) | 54 (44-67) | 55 (45-66) | 0.812 | 0.034 |
| Male (%) | 59/104 (56.7) | 28/52 (53.8) | 31/52 (59.6) | 0.692 | 0.117 |
| Weight (kg) | 83.9 (73.7-105.7) | 81.6 (71.9-98.5) | 86.8 (74.6-109.2) | 0.292 | 0.145 |
| Interventions |  |  |  |  |  |
| RRT use (1^st^ 24h) | 2/104 (1.9) | 1/52 (1.9) | 1/52 (1.9) | 1.000 | <0.001 |
| MV use (1^st^ 24h) | 39/104 (37.5) | 18/52 (34.6) | 21/52 (40.4) | 0.685 | 0.119 |
| Severity |  |  |  |  |  |
| SOFA score | 10 (7-12) | 10 (7-11) | 10 (8-12) | 0.606 | 0.070 |
| Comorbidities, n (%) |  |  |  |  |  |
| Cirrhosis | 2/104 (1.9) | 1/52 (1.9) | 1/52 (1.9) | 1.000 | <0.001 |
| Diabetes | 22/104 (21.2) | 12/52 (23.1) | 10/52 (19.2) | 0.810 | 0.094 |
| Vital signs |  |  |  |  |  |
| Heart rate (bpm) | 121 (104-146) | 120 (103-145) | 122 (104-148) | 0.785 | 0.044 |
| MAP (mmHg) | 61 (49-117) | 62 (52-111) | 60 (47-125) | 0.694 | 0.058 |
| Respiratory rate (bpm) | 28 (13-34) | 28 (12-34) | 28 (18-34) | 0.623 | 0.125 |
| Temperature (°C) | 36.7 (36.4-37.1) | 36.7 (36.4-37.1) | 36.7 (36.4-37.1) | 0.876 | 0.072 |
| Laboratory tests |  |  |  |  |  |
| pO2 (mmHg) | 97 (95-99) | 97 (94-99) | 97 (95-99) | 0.731 | 0.069 |
| Platelet (×10^9^/L) | 190 (128-293) | 204 (140-284) | 184 (112-309) | 0.489 | 0.031 |
| WBC (×10^9^/L) | 11.2 (8.3-16.1) | 11.4 (8.3-15.6) | 10.9 (8.7-16.6) | 0.696 | 0.087 |
| Albumin (g/dL) | 2.4 (1.9-2.9) | 2.4 (1.9-2.9) | 2.4 (1.9-2.9) | 0.807 | 0.044 |
| BUN (mg/dL) | 30 (15-53) | 25 (12-52) | 31 (23-53) | 0.382 | 0.023 |
| Creatinine (mg/dL) | 1.7 (0.8-3.3) | 1.5 (0.8-3.6) | 1.9 (1.1-3.1) | 0.351 | 0.020 |

PSM propensity score matching, Alb human serum albumin infusion, SMD standardized mean differences, RRT renal replacement therapy, MV mechanical ventilation, SOFA Sequential Organ Failure Assessment, MAP mean arterial pressure, pO2 partial pressure of oxygen, WBC white blood cell, BUN blood urea nitrogen

**Table S4: Baseline characteristics and SMD between treatment groups of acute pancreatitis patients from the MIMIC-IV database after PSM and IPTW matching**

| Covariates | IPTW | | | | PSM | | | |
| --- | --- | --- | --- | --- | --- | --- | --- | --- |
|  | non-Alb | Alb | SMD | *p* | non-Alb | Alb | SMD | *p* |
| N | 948 | 955 |  |  | 228 | 228 |  |  |
| Age | 58 (46-72) | 63 (50-76) | 0.160 | 0.206 | 60 (49-76) | 58 (48-72) | 0.100 | 0.301 |
| Male (%) | 545/948 (57.5) | 483/955 (50.6) | 0.138 | 0.275 | 130/228 (57.0) | 131/228 (57.5) | 0.009 | 1.000 |
| Weight (kg) | 81.0 (69.1-97.6) | 81.2 (71.5-107.7) | 0.106 | 0.307 | 82.1 (70.0-97.8) | 81.4 (71.2-99.5) | 0.016 | 0.546 |
| Admission period, n (%) |  |  | 0.060 | 0.581 |  |  | 0.072 | 0.502 |
| 2008-2013 | 608/948 (64.1) | 639/955 (67.0) |  |  | 143/228 (62.7) | 135/228 (59.2) |  |  |
| 2014-2019 | 341/948 (35.9) | 316/955 (33.0) |  |  | 85/228 (37.3) | 93/228 (40.8) |  |  |
| Interventions, n (%) |  |  |  |  |  |  |  |  |
| RRT use (1^st^ 24h) | 62/948(6.5) | 66/955 (6.9) | 0.018 | 0.838 | 25/228 (11.0) | 29/228 (12.7) | 0.054 | 0.664 |
| MV use (1^st^ 24h) | 321/948 (33.9) | 335/955 (35.1) | 0.027 | 0.804 | 116/228 (50.9) | 129/228 (56.6) | 0.115 | 0.260 |
| Severity |  |  |  |  |  |  |  |  |
| SOFA score | 5 (3-9) | 5 (1-9) | 0.036 | 0.610 | 8 (4-11) | 9 (5-13) | 0.232 | 0.019 |
| SAPS II score | 33 (23-45) | 35 (22-45) | 0.035 | 0.643 | 41 (31-53) | 43 (34-57) | 0.175 | 0.058 |
| Comorbidities, n (%) |  |  |  |  |  |  |  |  |
| CHF | 190/948 (20.0) | 271/955 (28.4) | 0.198 | 0.201 | 51/228 (22.4) | 40/228 (17.5) | 0.121 | 0.241 |
| COPD | 204/948 (21.5) | 179/955 (18.8) | 0.067 | 0.486 | 50/228 (21.9) | 53/228 (23.2) | 0.031 | 0.823 |
| Liver disease | 265/948 (27.9) | 245/955 (25.7) | 0.050 | 0.620 | 77/228 (33.8) | 93/228 (40.8) | 0.146 | 0.146 |
| Renal disease | 166/948 (17.5) | 239/955 (25.0) | 0.185 | 0.252 | 49/228 (21.5) | 46/228 (20.2) | 0.032 | 0.818 |
| Malignancy | 75/948 (7.9) | 87/955 (9.1) | 0.043 | 0.625 | 28/228 (12.3) | 29/228 (12.7) | 0.013 | 1.000 |
| Vital signs |  |  |  |  |  |  |  |  |
| Heart rate (bpm) | 93 (80-107) | 92 (85-107) | 0.082 | 0.566 | 100 (87-112) | 100 (86-112) | 0.006 | 0.830 |
| MAP (mmHg) | 81 (73-91) | 83 (73-94) | 0.132 | 0.319 | 80 (71-88) | 76 (71-86) | 0.113 | 0.323 |
| Respiratory rate (bpm) | 20 (17-24) | 21 (18-25) | 0.185 | 0.191 | 21 (18-25) | 21 (18-25) | 0.001 | 0.801 |
| Temperature (°C) | 36.9 (36.6-37.3) | 37.0 (36.7-37.2) | 0.040 | 0.715 | 36.9 (36.6-37.4) | 36.9 (36.6-37.2) | 0.046 | 0.389 |
| Laboratory tests |  |  |  |  |  |  |  |  |
| pH | 7.37 (7.29-7.43) | 7.35 (7.25-7.41) | 0.155 | 0.170 | 7.36 (7.27-7.42) | 7.36 (7.26-7.41) | 0.033 | 0.407 |
| pO2 (mmHg) | 81 (50-137) | 77 (52-157) | 0.022 | 0.982 | 88 (54-172) | 87 (57-167) | 0.013 | 0.807 |
| pCO2 (mmHg) | 39 (33-46) | 39 (34-43) | 0.014 | 0.780 | 39 (34-47) | 39 (33-44) | 0.050 | 0.755 |
| Lactate level (mmol/L) | 1.7 (1.2-2.7) | 2.0 (1.3-3.1) | 0.084 | 0.172 | 1.9 (1.4-3.3) | 2.1 (1.4-3.5) | 0.101 | 0.433 |
| Hemoglobin (×10^12^/L) | 11.2 (9.6-12.6) | 11.1 (9.5-13.9) | 0.158 | 0.366 | 11.0 (9.4-12.3) | 10.7 (9.0-12.6) | 0.035 | 0.387 |
| Platelet (×10^9^/L) | 185 (128-267) | 200 (144-265) | 0.012 | 0.393 | 180 (119-254) | 172 (117-264) | 0.043 | 0.623 |
| WBC (×10^9^/L) | 12.5 (8.6-17.2) | 12.9 (9.9-18.4) | 0.093 | 0.083 | 13.4 (9.3-19.0) | 13.0 (9.0-19.5) | 0.041 | 0.861 |
| Albumin (g/dL) | 3.1 (2.6-3.5) | 3.1 (2.7-3.7) | 0.116 | 0.458 | 2.9 (2.5-3.4) | 2.8 (2.4-3.3) | 0.186 | 0.085 |
| BUN (mg/dL) | 19 (12-35) | 21 (15-36) | 0.005 | 0.072 | 24 (15-41) | 26 (17-48) | 0.135 | 0.096 |
| Creatinine (mg/dL) | 1.0 (0.7-1.9) | 1.2 (0.8-1.7) | 0.033 | 0.325 | 1.2 (0.8-2.2) | 1.4 (0.9-2.6) | 0.063 | 0.157 |

SMD standardized mean differences, PSM propensity score matching, IPTW propensity score-based inverse probability of treatment weighting, Alb human serum albumin infusion, RRT renal replacement therapy, MV mechanical ventilation, SOFA Sequential Organ Failure Assessment, SAPS II Simplified Acute Physiology Score II, CHF congestive heart failure, COPD chronic obstructive pulmonary disease, MAP mean arterial pressure, pO2 partial pressure of oxygen, pCO2 partial pressure of carbon dioxide, WBC white blood cell, BUN blood urea nitrogen

**Table S5: Baseline characteristics of acute pancreatitis patients without missing data from the MIMIC-IV database**

| Covariates | MIMIC-IV (n=640) | | | | | | | | |
| --- | --- | --- | --- | --- | --- | --- | --- | --- | --- |
|  | All patients | | non-Alb | | Alb | | P value | SMD | |
| N | 640 | | 451 | | 189 | |  |  | |
| Age | 58 (46-71) | | 57 (45-70) | | 59 (50-73) | | 0.100 | 0.142 | |
| Male (%) | 373/640 (58.3) | | 262/451 (58.1) | | 111/189 (58.7) | | 0.951 | 0.013 | |
| Weight (kg) | 82.4 (70.0-100.0) | | 82.4 (70.0-99.4) | | 81.9 (71.0-100.3) | | 0.557 | 0.012 | |
| Admission period, n (%) |  | |  | |  | | 0.585 | 0.055 | |
| 2008-2013 | 388/640 (60.6) | | 277/451 (61.4) | | 111/189 (58.7) | |  |  | |
| 2014-2019 | 252/640 (39.4) | | 174/451 (38.6) | | 78/189 (41.3) | |  |  | |
| Interventions, n (%) |  | |  | |  | |  |  | |
| RRT use (1^st^ 24h) | 56/640 (8.8) | | 29/451 (6.4) | | 27/189 (14.3) | | 0.002 | 0.260 | |
| MV use (1^st^ 24h) | 276/640 (43.1) | | 163/451 (36.1) | | 113/189 (59.8) | | <0.001 | 0.487 | |
| Severity |  | |  | |  | |  |  | |
| SOFA score | 6 (3-10) | | 5 (3-9) | | 10 (6-13) | | <0.001 | 0.739 | |
| SAPS II score | 36 (26-49) | | 32 (24-44) | | 45 (36-58) | | <0.001 | 0.784 | |
| Comorbidities, n (%) |  | |  | |  | |  |  | |
| CHF | 123/640 (19.2) | | 92/451 (20.4) | | 31/189 (16.4) | | 0.289 | 0.103 | |
| COPD | 141/640 (22.0) | | 96/451 (21.3) | | 45/189 (23.8) | | 0.550 | 0.060 | |
| Liver disease | 204/640 (31.9) | | 125/451 (27.7) | | 79/189 (41.8) | | 0.001 | 0.299 | |
| Renal disease | 117/640 (18.3) | | 78/451 (17.3) | | 39/189 (20.6) | | 0.376 | 0.085 | |
| Malignancy | 55/640 (8.6) | | 31/451 (6.9) | | 24/189 (12.7) | | 0.025 | 0.197 | |
| Vital signs |  | |  | |  | |  |  | |
| Heart rate (bpm) | 96 (83-109) | | 95 (82-108) | | 101 (87-112) | | 0.003 | 0.280 | |
| MAP (mmHg) | 80 (72-91) | | 82 (74-92) | | 77 (71-86) | | <0.001 | 0.368 | |
| Respiratory rate (bpm) | 21 (18-24) | | 20 (18-24) | | 21 (18-25) | | 0.127 | 0.156 | |
| Temperature (°C) | 37.0 (36.7-37.4) | | 37.0 (36.7-37.5) | | 36.9 (36.6-37.3) | | 0.002 | 0.248 | |
| Laboratory tests |  | |  | |  | |  |  | |
| Lactate level (mmol/L) | 1.8 (1.2-2.9) | | 1.7 (1.2-2.6) | | 2.1 (1.4-3.5) | | <0.001 | 0.387 | |
| Hemoglobin (×10^12^/L) | 11.0 (9.5-12.6) | | 11.2 (9.7-12.7) | | 10.7 (9.2-12.6) | | 0.102 | 0.108 | |
| Platelet (×10^9^/L) | 180 (125-265) | | 185 (127-261) | | 173 (116-265) | | 0.242 | 0.047 | |
| WBC (×10^9^/L) | 12.6 (8.8-17.4) | | 12.4 (8.7-16.6) | | 13.1 (8.9-19.5) | | 0.042 | 0.207 | |
| Albumin (g/dL) | 3.0 (2.6-3.5) | | 3.1 (2.7-3.6) | | 2.8 (2.3-3.3) | | <0.001 | 0.474 | |
| BUN (mg/dL) | 21 (13-38) | | 18 (12-33) | | 27 (17-49) | | <0.001 | 0.330 | |
| Creatinine (mg/dL) | 1.1 (0.8-2.1) | | 1.0 (0.7-1.8) | | 1.5 (1.0-2.8) | | <0.001 | 0.218 | |
| Alb human serum albumin infusion, SMD standardized mean differences, RRT renal replacement therapy, MV mechanical ventilation, SOFA Sequential Organ Failure Assessment, SAPS II Simplified Acute Physiology Score II, CHF congestive heart failure, COPD chronic obstructive pulmonary disease, MAP mean arterial pressure, WBC white blood cell, BUN blood urea nitrogen | | | | | | | | | |
|  | | | | | | | | | |
| **Table S6: Effect of** **human serum albumin infusion on primary outcome in acute pancreatitis patients without missing data from the MIMIC-IV database before and after matching through multivariate Cox regressions** | | | | | | | | |  |
| Model | | NO.of  patients | | HR (95% CI) | | P Value | | |  |
| Multivariate Cox Model | | 640 | | 1.12 (0.70-1.78) | | 0.648 | | |  |
| PSM | | 378 | | 0.91 (0.58-1.43) | | 0.683 | | |  |
| IPTW | | 1,254 | | 1.03 (0.65-1.64) | | 0.893 | | |  |

HR hazard ratio, PSM Propensity score matching, IPTW inverse probability of treatment weighing

| **Table S7: Effect of human serum albumin infusion on primary outcome in acute pancreatitis patients with < 2.5 g/dL** **initial serum albumin from the MIMIC-IV database before and after matching through multivariate Cox regressions** | | | |
| --- | --- | --- | --- |
| Model | NO.of  patients | HR (95% CI) | P Value |
| Multivariate Cox Model | 175 | 1.78 (0.62-5.15) | 0.286 |
| PSM | 144 | 2.28 (0.69-7.61) | 0.182 |
| IPTW | 362 | 1.98 (0.68-5.74) | 0.209 |
| HR hazard ratio, PSM Propensity score matching, IPTW inverse probability of treatment weighing | | | |
|  | | | |
| **Table S8: Effect of human serum albumin infusion on primary outcome in acute pancreatitis patients with 2.5-3.0 g/dL initial serum albumin from the MIMIC-IV database before and after matching through multivariate Cox regressions** | | | |
| Model | NO.of  patients | HR (95% CI) | P Value |
| Multivariate Cox Model | 247 | 0.66 (0.20-2.23) | 0.508 |
| PSM | 128 | 0.59 (0.17-2.06) | 0.404 |
| IPTW | 534 | 1.06 (0.36-3.11) | 0.911 |
| HR hazard ratio, PSM Propensity score matching, IPTW inverse probability of treatment weighing | | | |
|  | | | |
| **Table S9: Effect of human serum albumin infusion on primary outcome in acute pancreatitis patients with 3.0-3.5 g/dL initial serum albumin from the MIMIC-IV database before and after matching through multivariate Cox regressions** | | | |
| Model | NO.of  patients | HR (95% CI) | P Value |
| Multivariate Cox Model | 250 | 8.17 (2.01-33.14) | 0.003 |
| PSM | 86 | 10.74 (1.80-64.18) | 0.009 |
| IPTW | 486 | 54.65 (6.54-456.91) | <0.001 |
| HR hazard ratio, PSM Propensity score matching, IPTW inverse probability of treatment weighing | | | |
|  | | | |
| **Table S10: Effect of human serum albumin infusion on primary outcome in acute pancreatitis patients with ≥3.5 g/dL initial serum albumin from the MIMIC-IV database before and after matching through multivariate Cox regressions** | | | |
| Model | NO.of  patients | HR (95% CI) | P Value |
| Multivariate Cox Model | 278 | 0.42 (0.13-1.34) | 0.141 |
| PSM | 98 | 0.29 (0.09-0.99) | 0.048 |
| IPTW | 529 | 0.40 (0.13-1.26) | 0.117 |
| HR hazard ratio, PSM Propensity score matching, IPTW inverse probability of treatment weighing | | | |

| **Table S11: Effect of human serum albumin infusion on primary outcome in acute pancreatitis patients with positive blood or peritoneal fluid bacterial culture from the MIMIC-IV database before and after matching through multivariate Cox regressions** | | | |
| --- | --- | --- | --- |
| Model | NO.of  patients | HR (95% CI) | P Value |
| Multivariate Cox Model | 161 | 1.36 (0.56-3.28) | 0.492 |
| PSM | 152 | 1.34 (0.51-3.53) | 0.559 |
| IPTW | 311 | 0.98 (0.43-2.25) | 0.968 |

HR hazard ratio, PSM Propensity score matching, IPTW inverse probability of treatment weighing

| **Table S12: Effect of human serum albumin infusion on primary outcome in acute pancreatitis patients with ≤ 100 g infusion from the MIMIC-IV database before and after matching through multivariate Cox regressions** | | | |
| --- | --- | --- | --- |
| Model | NO.of  patients | HR (95% CI) | P Value |
| Multivariate Cox Model | 861 | 1.40 (0.87-2.26) | 0.166 |
| PSM | 278 | 1.57 (0.83-2.99) | 0.169 |
| IPTW | 1,682 | 1.74 (1.02-2.95) | 0.040 |
| HR hazard ratio, PSM Propensity score matching, IPTW inverse probability of treatment weighing | | | |
|  | | | |
| **Table S13: Effect of human serum albumin infusion on primary outcome in acute pancreatitis patients with > 100 g infusion from the MIMIC-IV database before and after matching through multivariate Cox regressions** | | | |
| Model | NO.of  patients | HR (95% CI) | P Value |
| Multivariate Cox Model | 811 | 0.70 (0.40-1.24) | 0.219 |
| PSM | 178 | 0.65 (0.34-1.22) | 0.182 |
| IPTW | 1,550 | 0.98 (0.47-2.02) | 0.949 |

HR hazard ratio, PSM Propensity score matching, IPTW inverse probability of treatment weighing

| **Table S14: Effect of early (within 72 hours after ICU admission) human serum albumin infusion on primary outcome in acute pancreatitis patients from the MIMIC-IV database before and after matching through multivariate Cox regressions** | | | |
| --- | --- | --- | --- |
| Model | NO.of  patients | HR (95% CI) | P Value |
| Multivariate Cox Model | 872 | 0.88 (0.54-1.42) | 0.604 |
| PSM | 300 | 0.74 (0.46-1.20) | 0.220 |
| IPTW | 1,655 | 1.20 (0.68-2.13) | 0.529 |

HR hazard ratio, PSM Propensity score matching, IPTW inverse probability of treatment weighing


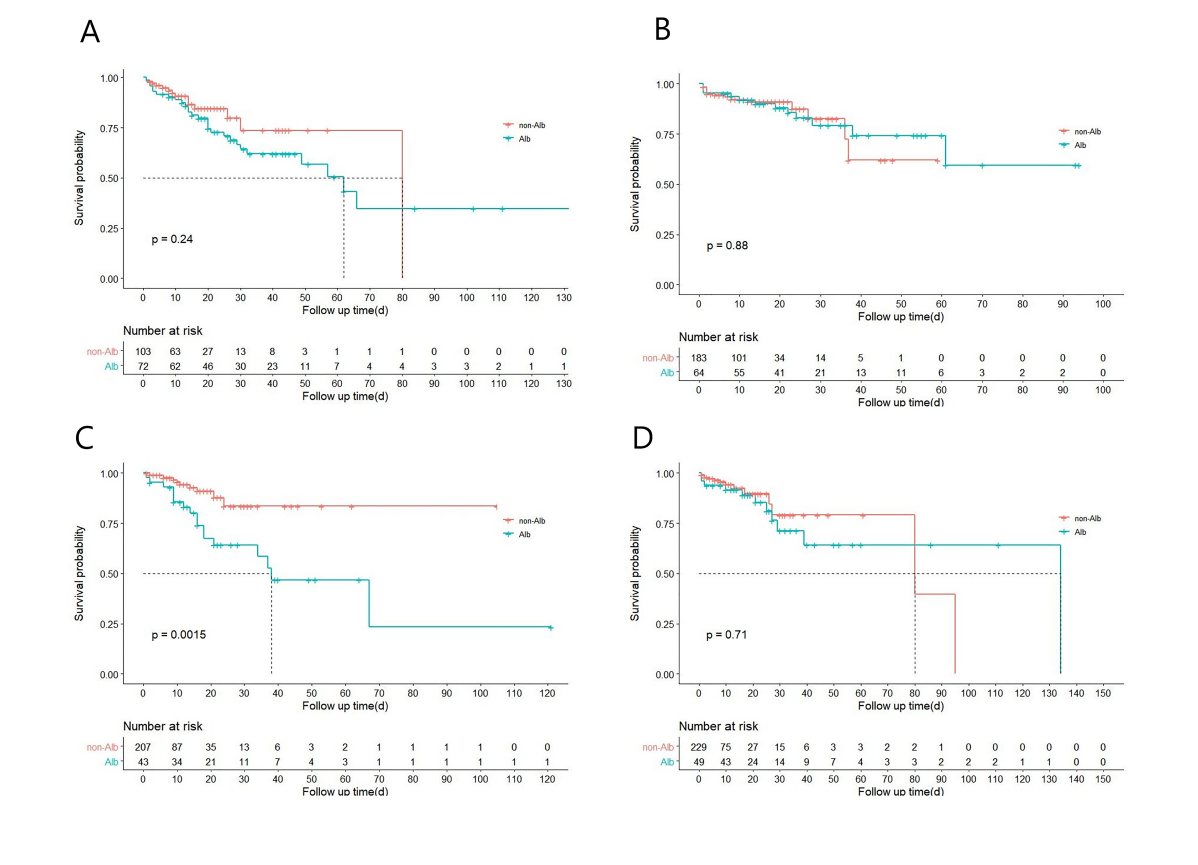


**Figure S1:** **Survival curves of hospital mortality in acute pancreatitis patients between treatment groups with different initial serum albumin levels from the MIMIC-IV database. A < 2.5 g/dL group. B 2.5-3.0 g/dL group. C 3.0-3.5 g/dL group. D ≥ 3.5 g/dL group**


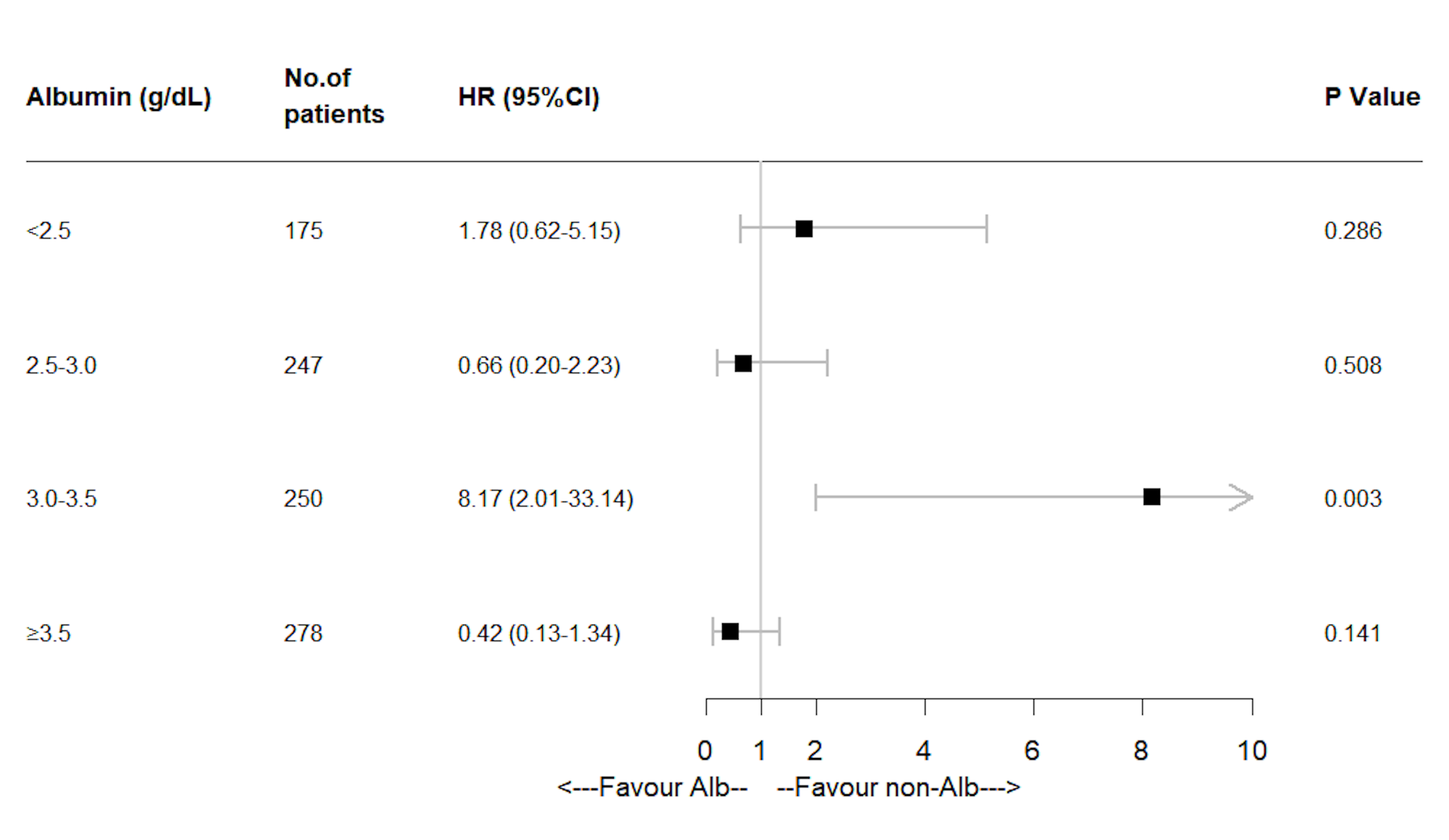


**Figure S2: Effect of human serum albumin infusion on primary outcome in acute pancreatitis patients with different initial serum albumin levels from the MIMIC-IV database through multivariate Cox regressions**


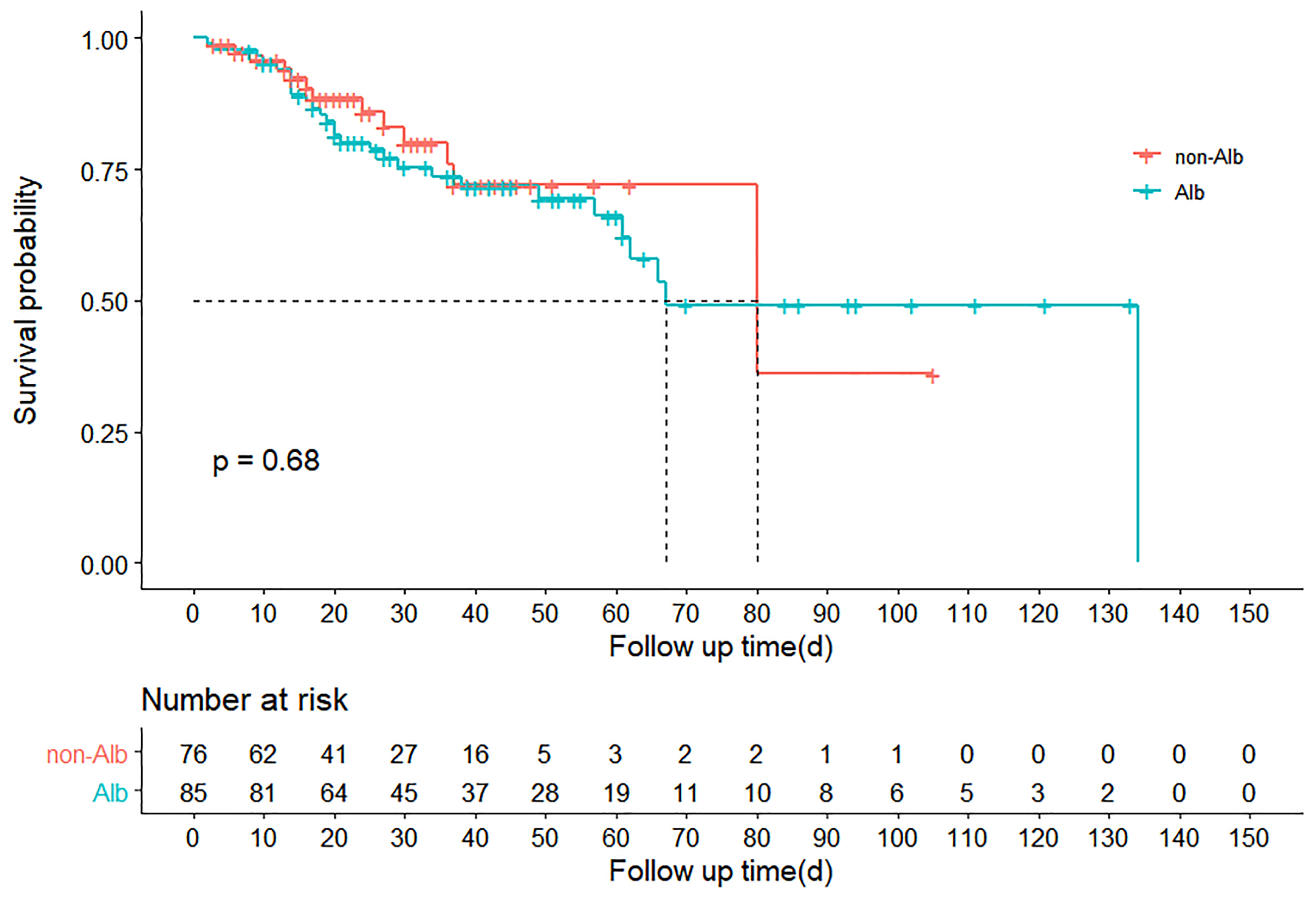


**Figure S3: Survival curves of hospital mortality in acute pancreatitis patients between treatment groups with positive blood or peritoneal fluid bacterial culture from the MIMIC-IV database**

**
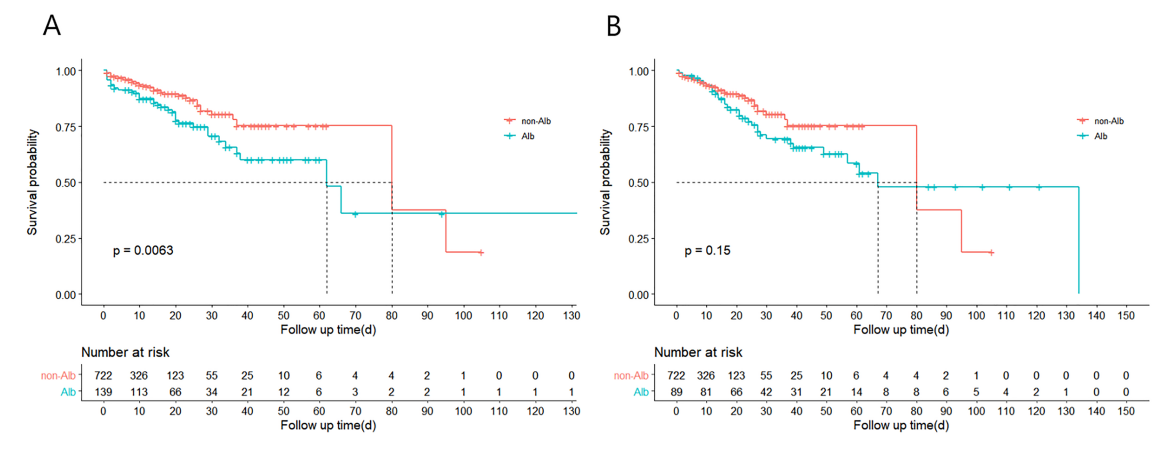
**

**Figure S4: Survival curves of hospital mortality in acute pancreatitis patients between treatment groups with different total albumin infusion doses from the MIMIC-IV database. A < 100 g group. B ≥ 100 g group**

**
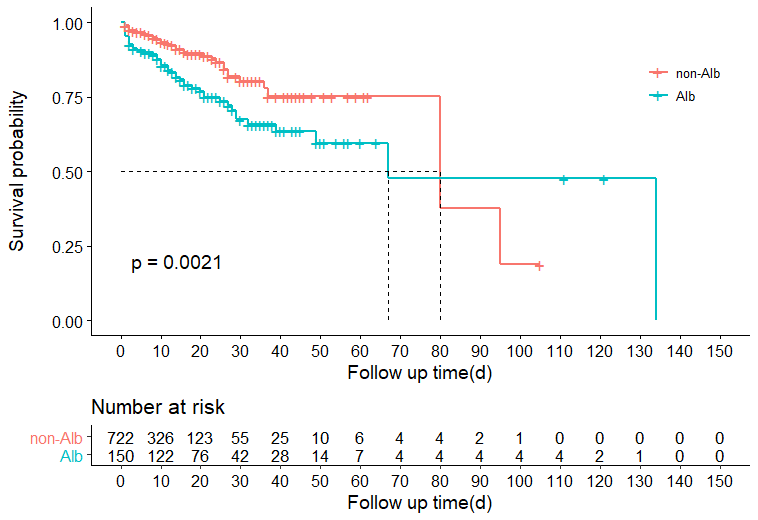
**

**Figure S5: Survival curves of hospital mortality in acute pancreatitis patients between early human serum albumin infusion cohort (within 72 hours after ICU admission) and control group from the MIMIC-IV database**


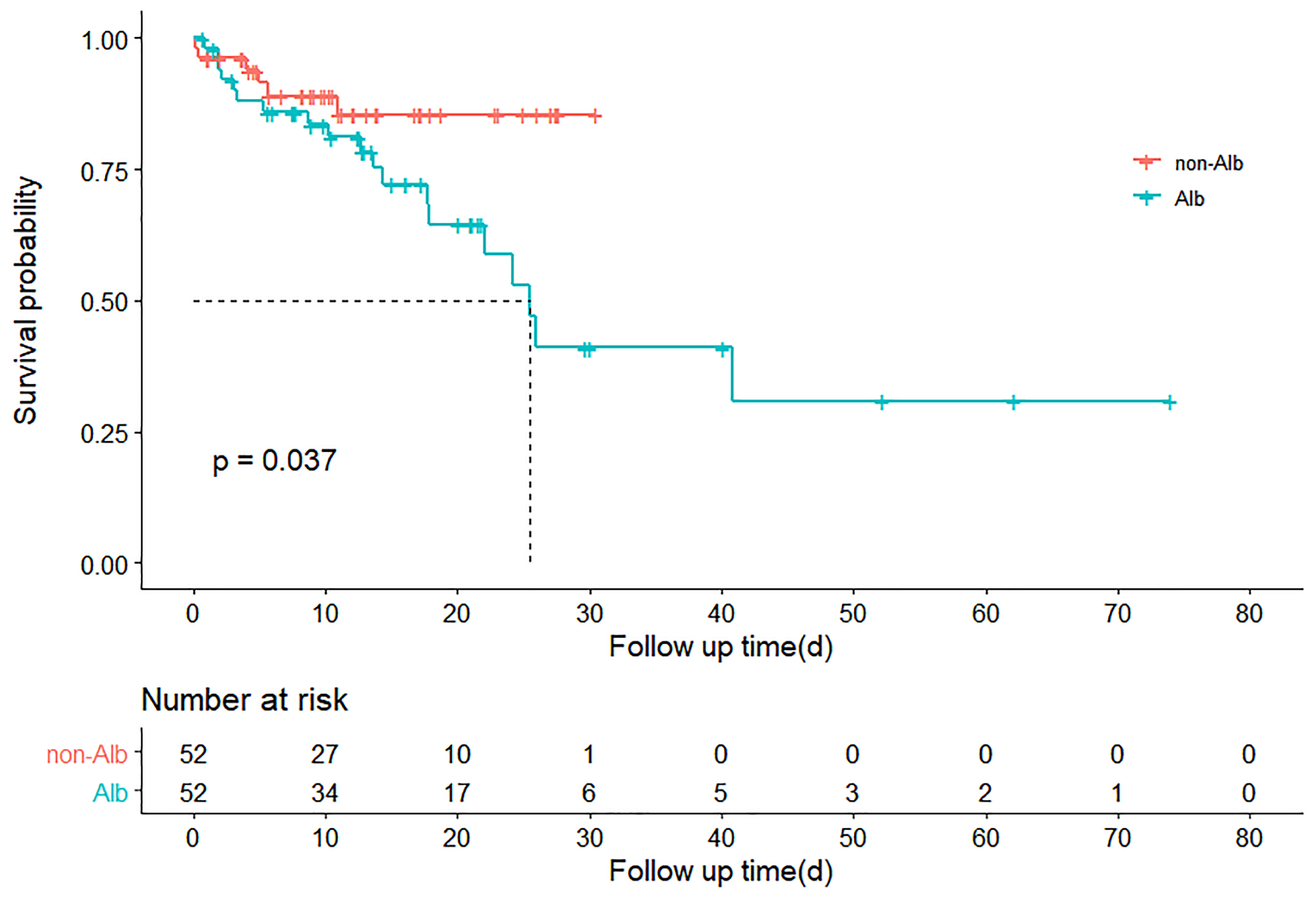


**Figure S6: Survival curves of hospital mortality in acute pancreatitis patients between treatment groups from the eICU database after propensity score matching**


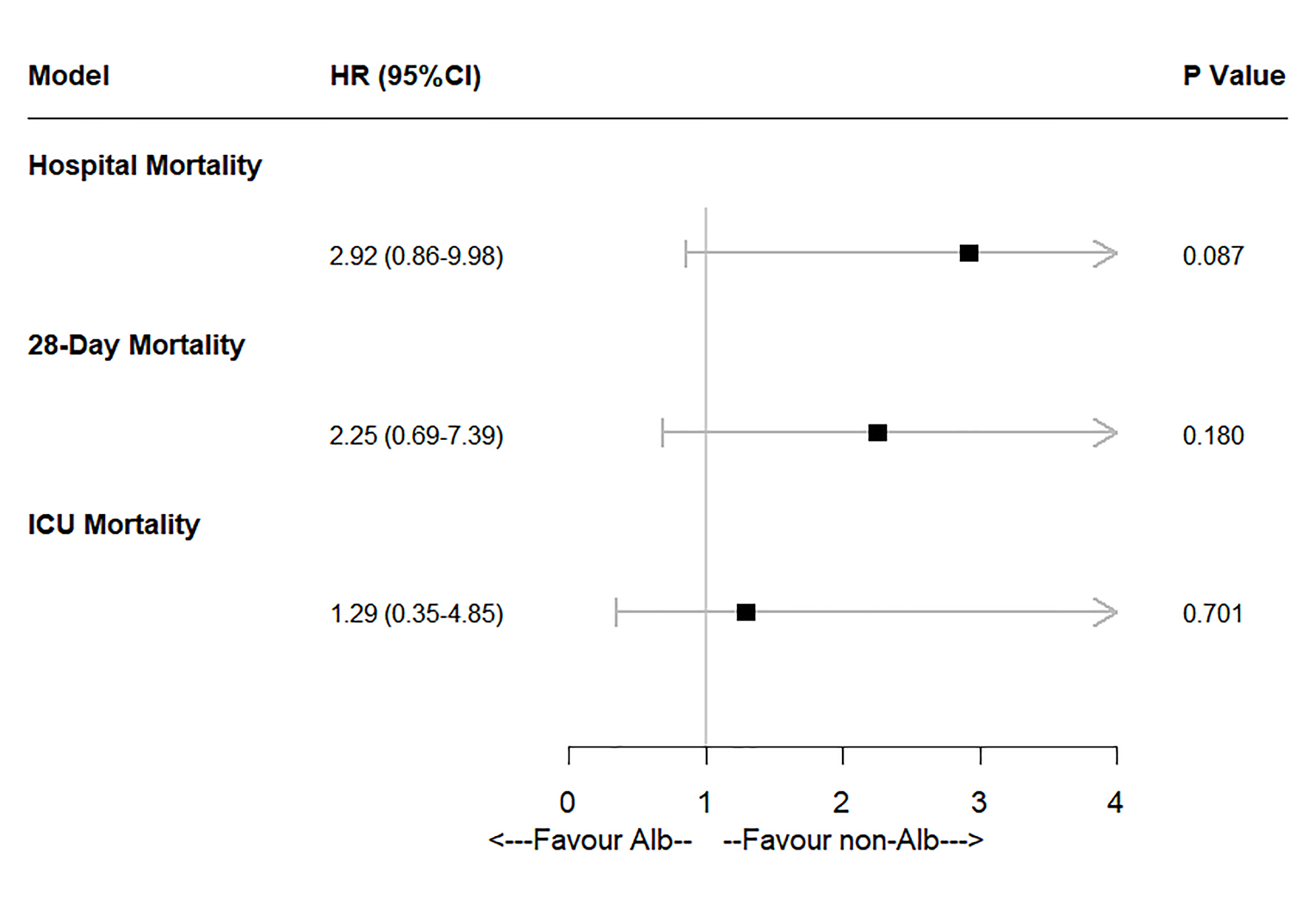


**Figure S7: Effect of human serum albumin infusion on multiple hospital outcomes in acute pancreatitis patients from the eICU database after propensity score matching through multivariate Cox regressions**
